# Comparative study of plasmonic antennas fabricated by electron beam and focused ion beam lithography

**DOI:** 10.1038/s41598-018-28037-1

**Published:** 2018-06-25

**Authors:** Michal Horák, Kristýna Bukvišová, Vojtěch Švarc, Jiří Jaskowiec, Vlastimil Křápek, Tomáš Šikola

**Affiliations:** 1 Central European Institute of Technology, Brno University of Technology, Purkyňova 123, 612 00, Brno, Czech Republic; 2 Institute of Physical Engineering, Brno University of Technology, Technická 2, 616 69, Brno, Czech Republic

## Abstract

We present a comparative study of plasmonic antennas fabricated by electron beam lithography and direct focused ion beam milling. We have investigated optical and structural properties and chemical composition of gold disc-shaped plasmonic antennas on a silicon nitride membrane fabricated by both methods to identify their advantages and disadvantages. Plasmonic antennas were characterized using transmission electron microscopy including electron energy loss spectroscopy and energy dispersive X-ray spectroscopy, and atomic force microscopy. We have found stronger plasmonic response with better field confinement in the antennas fabricated by electron beam lithography, which is attributed to their better structural quality, homogeneous thickness, and only moderate contamination mostly of organic nature. Plasmonic antennas fabricated by focused ion beam lithography feature weaker plasmonic response, lower structural quality with pronounced thickness fluctuations, and strong contamination, both organic and inorganic, including implanted ions from the focused beam. While both techniques are suitable for the fabrication of plasmonic antennas, electron beam lithography shall be prioritized over focused ion beam lithography due to better quality and performance of its products.

## Introduction

Localized surface plasmons (LSP) are collective oscillations of free electrons in metallic nanostructures (plasmonic antennas) coupled to the local electromagnetic field. A characteristic feature of LSP is a strong enhancement of electromagnetic field within the surrounding dielectric together with its confinement on the subwavelength scale. It can be utilized to control various optical processes in the visible and near infrared spectral region even below the free space diffraction limit^1^. This feature is utilized in numerous applications^2,3^. Properties of LSP can be tuned via engineering the size and shape of plasmonic antennas, or dielectric environment surrounding antennas^4^. Mapping of LSP resonances in metallic nanostructures with high spatial and energy resolution is necessary to understand their optical properties. Electron energy loss spectroscopy (EELS) is a technique utilizing an electron beam that interacts with the metallic nanoparticle and excites the LSP resonances^5–7^. In consequence, the energy of some electrons decreases by the characteristic energy of a plasmon resonance, which is then observable in the energy-dispersed spectra of transmitted electrons. Spatially-resolved EEL spectra further provide (relative) intensity of the near electric field of a LSP resonance projected to the trajectory of the electron beam. EELS tomography then provides a three-dimensional reconstruction of the plasmonic near field^8,9^. Additionally, EELS allows to determine the thickness of the sample^10,11^.

**Figure 1:**
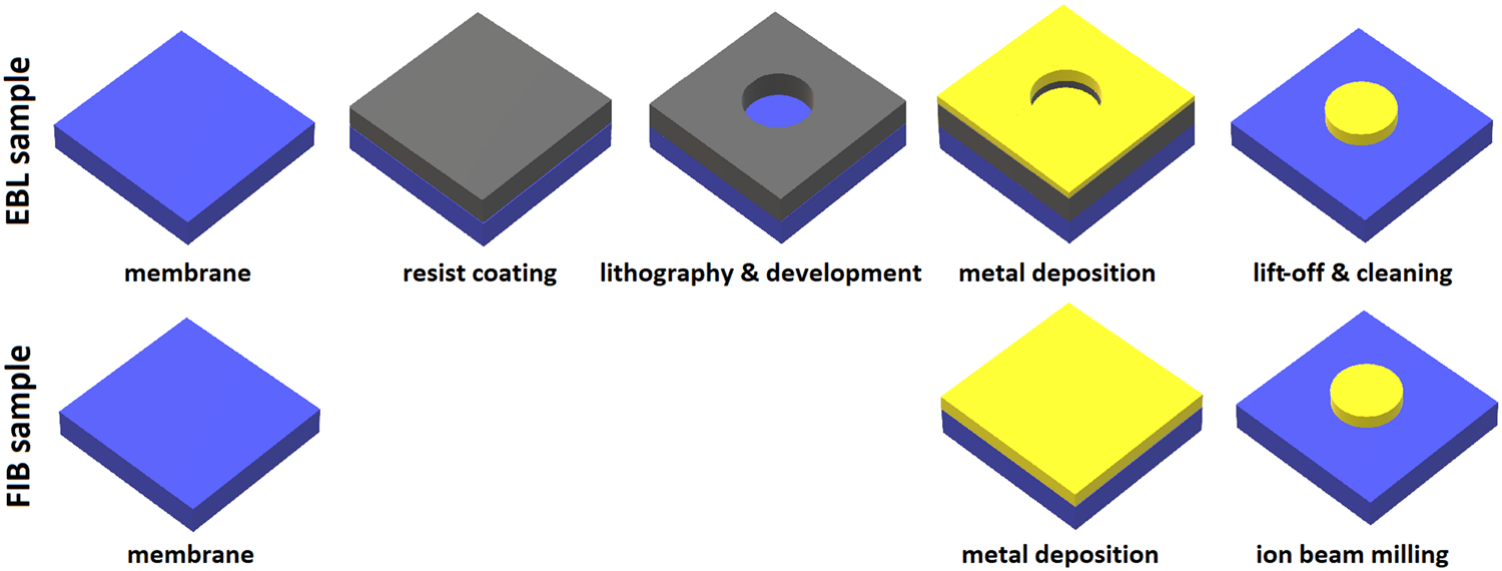
Schematic overview of individual steps in the fabrication process of the EBL and the FIB antennas.

Plasmonic antennas are often fabricated by electron beam lithography (EBL)^12^ or using focused ion beam (FIB) lithography^13^. The EBL process consists of following steps: (i) deposition of a resist sensitive to electron beam on the substrate, (ii) exposition of the resists to the electron beam and development of the resist (removal of exposed or unexposed regions of the resist), (iii) deposition of a thin layer of metal, and (iv) removal of the remaining resist covered by the redundant metal (lift-off) and final cleaning of the sample. FIB lithography is more straightforward, consisting of two steps: (i) deposition of a thin layer of metal and (ii) exposition to a focused ion beam that locally sputters off the metal (see Fig. 1). Both the EBL process and the FIB milling are capable to create sub-10-nm structures^14,15^.

Naturally, both fabrication techniques have their advantages and disadvantages. In EBL, final structures can be contaminated by the residual resist or solvents. Lift-off can exert a pronounced mechanical force that can damage the fabricated structure or even the substrate, which is particularly dangerous for thin membranes used as the substrate. In FIB, large-area mechanical effects are absent but the focused ion beam can damage the sample locally. Redeposition of sputtered-off metal atoms can reduce the quality of products of the technique. There are no chemicals involved in the process but the ions in the milling beam and atoms sputtered from the metal and substrate can still contaminate the fabricated structures. EBL consists of more steps but is more suitable for large area lithography as the exposition, the only local process, is considerably faster than for FIB. On the other hand, FIB is faster when only single antenna or a small field of antennas is being fabricated. For example, considering optimal parameters for FIB and EBL (see the Methods section), FIB milling of one antenna inside a 2 × 2 μm^2^ metal-free square takes typically 2 minutes. Array of 50 × 50 antennas is fabricated in 84 hours (3.5 days). On the other hand, in EBL the length of chemical processes (resist coating, development, and lift off) is independent on the number of fabricated antennas and takes typically 6 hours. Electron beam exposition of one antenna lasts far below 1 second; the array of 50 × 50 antennas is exposed in 5 seconds. FIB enables fabrication of single crystalline plasmonic antennas when a chemically grown single crystal gold flake transferred onto a substrate is used as the pristine material instead of sputtered or evaporated polycrystalline thin film^16,17^. The quality of lithographically fabricated plasmonic antennas can be further enhanced by annealing which enlarges the grains and accordingly reduces the number of grain boundaries so the behavior of nanostructures is closer to a single crystal^18,19^. It is clear that suitability of both methods depends on specific task and experimental comparison of the structures fabricated by both methods is of high importance for the judicious selection of the optimal method. We note that the subject is relevant for a wide range of nanostructures beyond plasmonic antennas, such as biosensors^20^ or metasurfaces^21^.

In our contribution, we present a comparative study of gold plasmonic disc-shaped antennas fabricated by EBL and FIB lithography with gallium ions. Fabricated antennas were characterized using the transmission electron microscopy (TEM) including chemical analysis by energy dispersive X-ray spectroscopy (EDS) and characterization of LSP resonances and thickness measurement by EELS, and using the atomic force microscopy (AFM) to complement information about the morphology of the structures. Spectral and spatial characteristics of LSP resonances obtained from experiment were compared with numerical simulations with the MNPBEM toolbox^22^ which relies on the boundary element method (BEM) approach^23,24^.

## Results

We have studied in detail four individual disc-shaped gold plasmonic antennas: two series prepared by both EBL and FIB consisting of antennas with the designed diameter of 120 and 140 nm and with the designed height of 25 nm. After their fabrication, several characterization methods have been applied. Some of those methods can modify the samples. In particular, a hydrocarbon contamination is developing during measurements in electron microscopes using high beam current (i.e., EELS and EDS)^25,26^. For this reason, the characterization methods have been applied in specific order for all investigated antennas: EELS, EDS, AFM. As we consider possible contamination to be a part of PA functionality, no specific cleaning steps have been applied between individual measurements. In particular, no plasma cleaning has been applied prior to EELS measurements, although it is often used to remove the hydrocarbon contamination.

**Figure 2:**
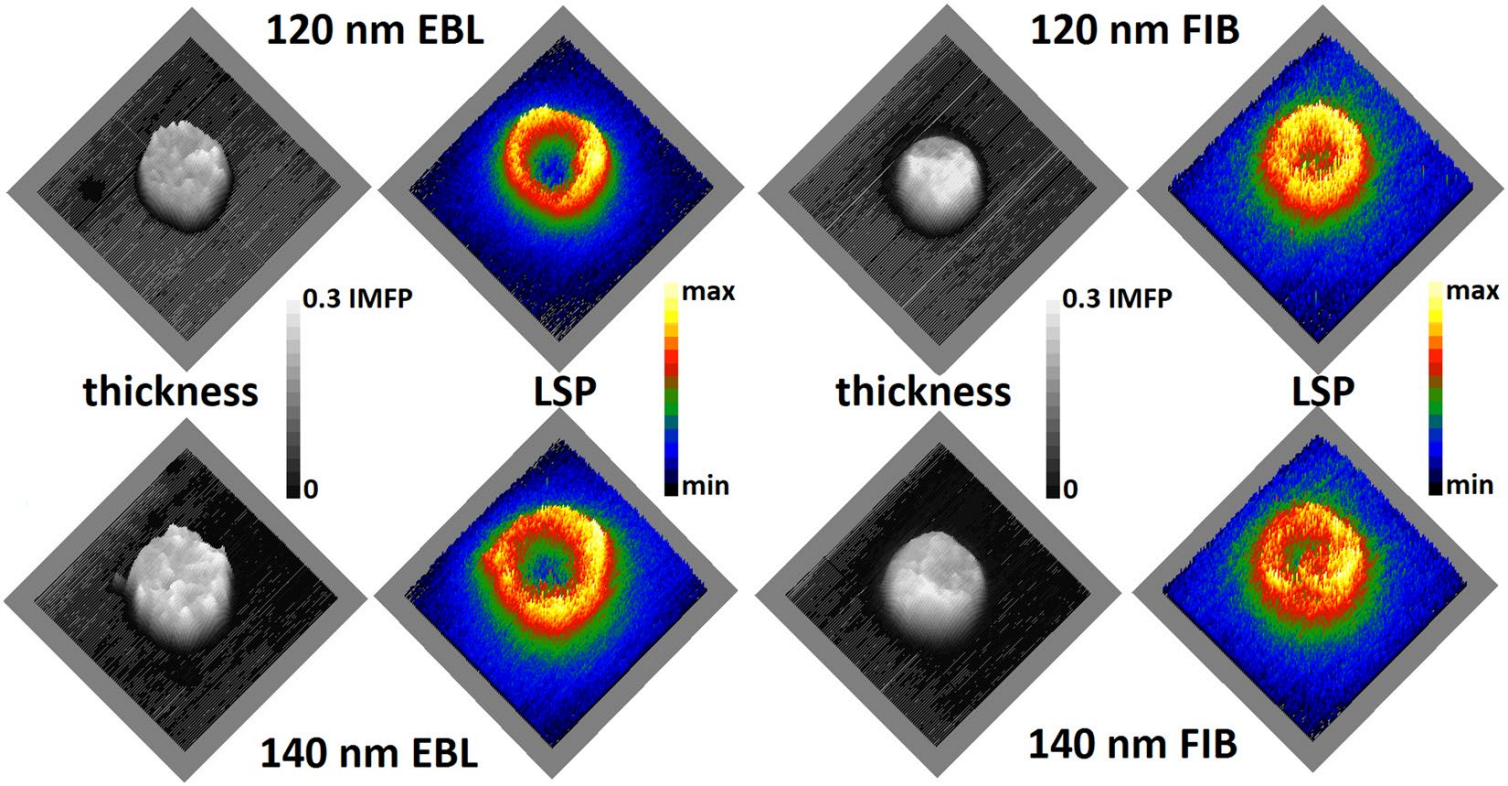
Thickness and LSP resonance mapping by EELS. 3D morphology of the antennas represented by the relative thickness (thickness in units of inelastic mean free path, IMFP) measured by EELS (left, grayscale) and EEL intensity maps showing the spatial distribution of LSP resonance at (1.60 ± 0.05) eV for 120 nm antennas and (1.40 ± 0.05) eV for 140 nm antennas (right, color scale). The size of all micrographs is 300 × 300 nm^2^.

**Figure 3:**
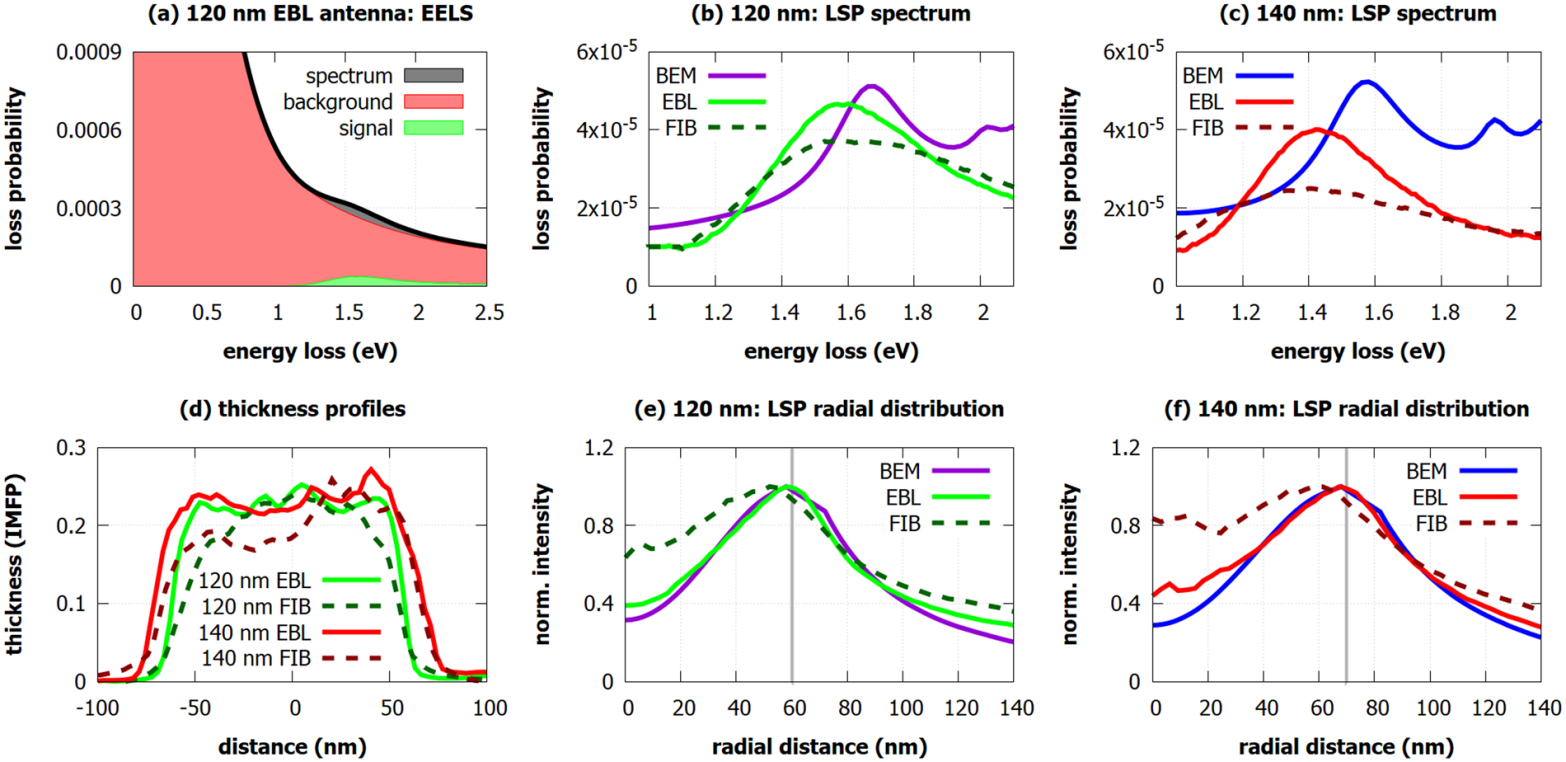
EELS measurement of LSP resonances. (**a**) Typical raw low-loss EEL spectrum (black) decomposed into background including zero-loss peak (red) and the signal corresponding to the LSP resonance (green) for the 120-nm EBL antenna. (**b**,**c**) Loss probability related to LSP measured by EELS in the EBL and the FIB antennas together with the calculated values obtained by BEM for 120-nm (**b**) and 140-nm (**c**) antennas. (**d**) Thickness profiles (thickness in units of inelastic mean free path, IMFP) of the antennas determined by EELS. We note that EBL antennas have slightly sharper edges. (**e**,**f**) The radial distribution of the LSP-related loss probability (at the energy of its maximum) for 120-nm (**e**) and 140-nm (**f**) antennas measured by EELS for EBL and FIB antennas and calculated by BEM. Antennas have their center at 0 nm and the edge of antennas is marked by the grey line.

The three-dimensional (3D) morphology of the antennas measured by EELS in terms of relative thickness is shown in Fig. 2. The average relative thickness is (0.23 ± 0.05) for 120-nm EBL antenna, (0.20 ± 0.07) for 120-nm FIB antenna, (0.23 ± 0.05) for 140-nm EBL antenna, and (0.20 ± 0.06) for 140-nm FIB antenna, see also Fig. 3d. The relative thickness is proportional to the absolute thickness of the antenna with the inelastic mean free path (IMFP) as the constant of proportionality. The IMFP in gold for the actual parameters of the electron beam (electron energy of 300 keV and collection semi-angle of 20.5 mrad) calculated using the software package EELSTools by D. Mitchell^27^ applying the algorithm of K. Iakoubovskii *et al*.^11^ equals to 113 nm. Consequently, the absolute thickness of the antennas is (23 ± 7) nm for FIB antennas and about (26 ± 6) nm for EBL antennas, which is in a good agreement with the desired value of 25 nm. Diameters of fabricated antennas measured from TEM micrographs indicate good agreement between the designed and actual value. They read (125 ± 5) nm for 120-nm EBL antenna, (122 ± 6) nm for 120-nm FIB antenna, (140 ± 6) nm for 140-nm EBL antenna, and (142 ± 7) nm for 140-nm FIB antenna.

There is a clear structural difference between EBL and FIB antennas. EBL antennas have an irregular shape and their thickness varies rapidly with the position as the gold layer is polycrystalline with the grain size of about 20 nm. FIB antennas are seemingly more uniform in the thickness and their edges are smooth and featureless. Nevertheless, a closer inspection (see Fig. 3d) reveals even more pronounced (i.e. with larger amplitude) but smoother (i.e. with reduced slope) variations of the thickness with the magnitude of nearly 10 nm. The flat boundary is mainly caused by two reasons. First, the metallic layer is deposited on the flat substrate for FIB but on the patterned resist for EBL forming a flat layer in the former case and frayed edges in the latter case. Second, the ion beam has a Gaussian-like profile which leads to smoothing of the edges. Additionally, the ion beam may result into amorphization and recrystallization of gold, smoothening its surface^28^. Finally, redeposition of sputtered-off gold may contribute to the smoothness of the gold surface and might be also partially responsible for the large magnitude of the thickness variation.

Next, EEL spectra were processed to obtain the loss probability related to LSP resonances. This included zero-loss peak (ZLP) and background subtraction. The process is illustrated in Fig. 3a showing the unprocessed low-loss part of EEL spectrum decomposed into the contribution of ZLP and background, and signal corresponding to the LSP resonance for the 120-nm EBL antenna. Extracted EEL signal corresponding to the LSP resonance is shown in Fig. 3b for 120-nm antennas and in Fig. 3c for 140-nm antennas. Within the spectra we resolve single broad peak corresponding to the dipole LSP mode. This assignment is further supported by the results of numeric simulation, also shown in Fig. 3b,c. It is also in agreement with prior investigations of plasmonic disc-shaped antennas^29^. The second peak in the calculated spectra corresponds to the quadrupole LSP mode. Due to its low intensity and instrumental broadening, related to the energy width of the impinging electron beam with the full-width at half-maximum (FWHM) of about 0.18 eV, this mode is not resolvable in the experimental data. The simulation predicts the energy of the dipole LSP mode of 1.66 eV for 120-nm antennas and 1.59 eV for 140-nm antennas. Measured LSP resonance energy is (1.56 ± 0.09) eV for 120-nm EBL antenna, (1.62 ± 0.09) eV for 120-nm FIB antenna, (1.43 ± 0.09) eV for 140-nm EBL antenna, and (1.41 ± 0.09) eV for 140-nm FIB antenna. The measured energies are thus systematically red-shifted from the calculated energies, although the magnitude of the shift is rather minor and ranges between 0.04 eV and 0.18 eV. Such a red-shift can be attributed to the deviations of the actual antenna shape from the ideal disc, inhomogeneous thickness of antennas, modification of the dielectric function of gold due to grain boundaries and polycrystallinity of the antennas^30,31^, or effects induced by the contamination^32^ and Ti adhesion layer^33,34^.

If we compare EBL and FIB antennas, we observe more intense peak for EBL antennas. Figure 2 shows the spatial distribution of the LSP resonance in the form of intensity maps at the energy of (1.60 ± 0.05) eV for 120-nm antennas and (1.40 ± 0.05) eV for 140-nm antennas. The radial dependences of the intensities averaged over the polar coordinate are shown in Fig. 3(e,f). The maximal intensity of LSP resonance is confined to sharp features on the edges of EBL antennas. In the case of FIB antennas, LSP resonance is less confined to the edges of the structures and the EEL intensity maps are blurred, which can be caused, for example, by thickness fluctuation or contamination.

**Figure 4:**
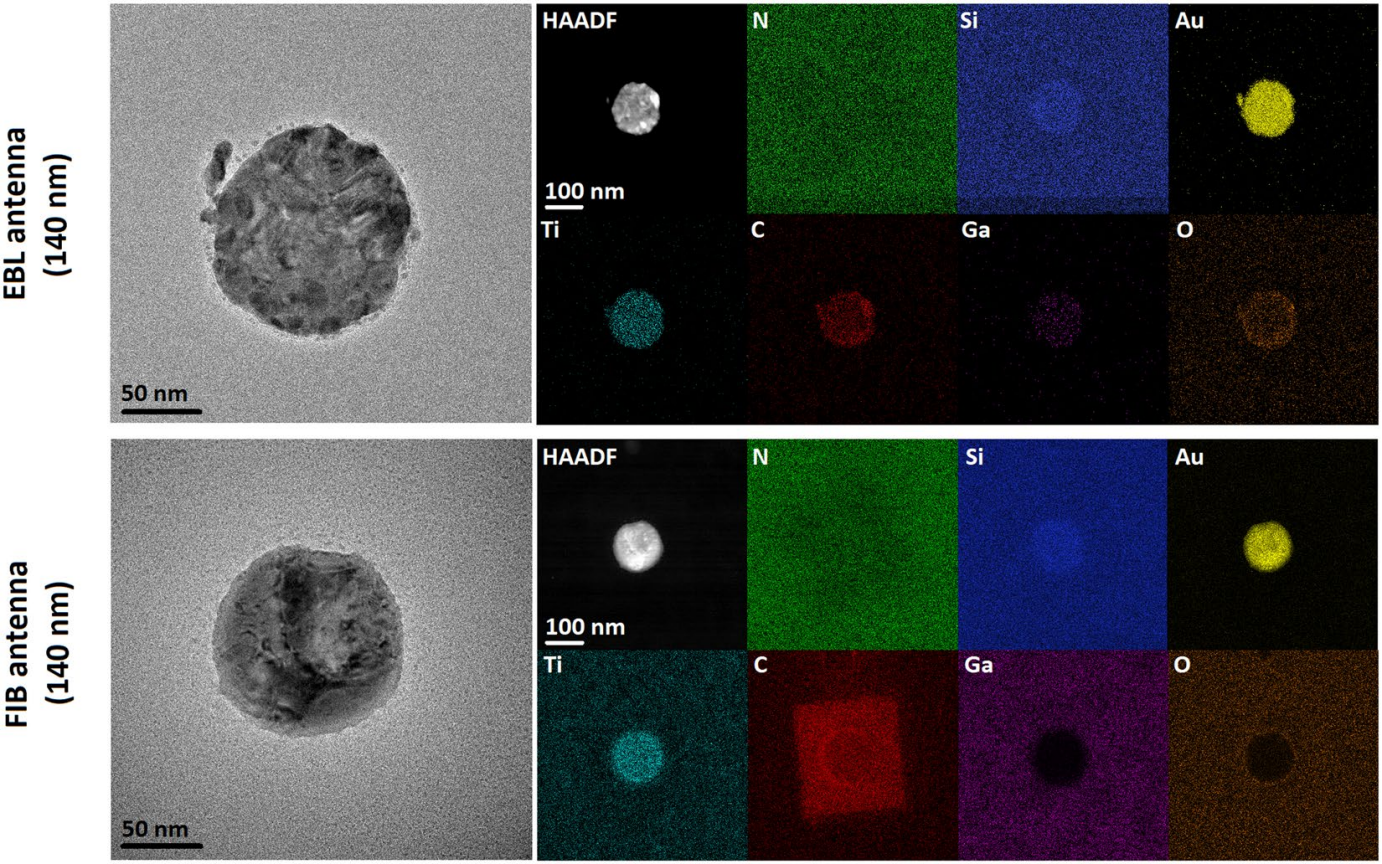
TEM micrographs and elemental maps showing chemical composition of 140-nm antennas. Left: TEM bright field micrographs of 140-nm EBL and FIB antennas. Right: HAADF image of the antennas presenting the distribution of heavy elements and spatial maps of element-specific X-ray intensity obtained from EDS for N, Si, Au, Ti, C, Ga, and O.

Following the EELS measurements we have analyzed the chemical composition of the antennas, including their possible contamination. To this end we performed EDS analysis, which enables detection of all elements heavier than Be in one measurement. We have detected and further focused on the following chemical elements: Si and N constituting the membrane, Au and Ti constituting the antenna and the adhesion layer, respectively, Ga that forms the ion beam in FIB and can be implanted into the sample, C and O as frequent contaminants. Spatial distribution of the intensity of characteristic X-ray radiation for these elements is shown in Fig. 4 for both FIB and EBL antennas with the diameter of 140 nm together with the bright field TEM and high-angle annular dark field (HAADF) micrographs. We distinguish in total three areas with different chemical composition, denoted as Area 1 to 3 in the following. Area 1 corresponds to the antenna and Areas 2 and 3 to its surrounding exposed and unexposed to the electron beam during EELS measurements. We note that the composition of Areas 2 and 3 is almost identical with the exception of carbon, which has a stronger presence in the Area 2 (exposed to the electron beam) in the case of FIB antenna (see intense red square in Fig. 4). The distribution of individual elements within each Area is homogeneous. The average relative atomic rate of each element for each Area is given in Table 1. As the thickness of different Areas is not identical, we note that the atomic rates between different Areas are not directly comparable but have to be rescaled to the same thickness.

**Table 1:** Chemical composition of the membranes with EBL and FIB antenna in atomic percents determined by EDS after the EELS measurement. The composition is averaged over one of three areas with homogeneous chemical compositions: Area 1 corresponds to the antenna, while Areas 2 and 3 corresponds to their surrounding exposed and unexposed to the electron beam during EELS measurements, respectively.

	EBL antenna			FIB antenna		
	Area 1	Area 2	Area 3	Area 1	Area 2	Area 3
Au	(18 ± 3)%	below 1%	below 1%	(16 ± 3)%	below 1%	below 1%
Ti	(4 ± 1)%	below 1%	below 1%	(4 ± 1)%	(2 ± 1)%	(2 ± 1)%
Si	(35 ± 5)%	(55 ± 5)%	(55 ± 5)%	(32 ± 5)%	(40 ± 5)%	(45 ± 5)%
N	(20 ± 3)%	(35 ± 5)%	(35 ± 5)%	(17 ± 3)%	(25 ± 5)%	(33 ± 5)%
C	(13 ± 3)%	(4 ± 2)%	(4 ± 2)%	(25 ± 5)%	(25 ± 5)%	(8 ± 3)%
O	(6 ± 2)%	(4 ± 2)%	(4 ± 2)%	(5 ± 2)%	(7 ± 2)%	(7 ± 2)%
Ga	below 1%	below 1%	below 1%	below 1%	(3 ± 1)%	(3 ± 1)%

Both Si and N exhibit almost identical X-ray intensity over all Areas for both FIB and EBL antennas. Slightly increased intensity of Si below the antennas (Area 1) is an artifact related to the secondary emission of X-rays in the silicon-based EDS detector associated with larger thickness and density of this Area. Average stoichiometry of SiN_x_ membrane (neglecting Area 1) reads Si(3)N(2.0 ± 0.6). Verification measurement by EELS resulted into average stoichiometry Si(3)N(1.8 ± 0.3) which is in a very good agreement with EDS. Gold is present only at the antennas (Area 1), underpinning reliability of both fabrication techniques. In general, EDS is not accurate enough to assess the thickness of the gold layer. It is nevertheless illustrative to provide a rough estimate. The atomic rate of Au is 0.18 ± 0.03 and 0.16 ± 0.03 in the EBL and FIB antenna, respectively. These values are equal within the experimental error. The atomic rates of Si and N are listed in Table 1. We now rescale the atomic rates to the volume rates using covalent radii of N, Si, and Au reading 71 pm, 116 pm, and 124 pm^35^. Considering the thickness of the SiN_x_ membrane of 50 nm, we obtain rough estimates of the thickness of the gold layer of 28 nm and 27 nm for the EBL and FIB antenna, respectively, in a good agreement with the designed thickness of 25 nm and EELS measurements of 23–26 nm.

Ti is present in the Area 1 for EBL antenna but in all Areas for FIB antenna. Apparently, FIB lithography was unable to fully remove Ti from the desired Areas as the sputter rate of Ti is much smaller than that of Au. Estimated thickness of the Ti contamination layer is 1–2 nm. Ga ions have been implanted to the Areas 2 and 3 (i.e. into the antenna surrounding) for FIB antennas. The atomic rate of Ga reads 0.03 ± 0.01. Importantly, no Ga contamination is found directly above or inside antennas (i.e. in Area 1), suggesting its rather limited influence on the plasmonic response of the antennas.

Finally, C and O are present as usual contaminants due to several reasons. EBL antenna indicates larger contamination in Area 1 (i.e., at the position of the antenna) where the atomic rate of C reads 0.13 ± 0.03 and the atomic rate of O is 0.06 ± 0.02. This is probably the result of incomplete resist removal during the development of lithographic pattern. Antenna surrounding (Areas 2 and 3) indicates low level of C and O contamination with the atomic rate of both elements of 0.04 ± 0.02. Such low level of contamination refers to usual contamination of the sample when it is exposed to air. FIB antenna indicates much higher C and O contamination. The sample has been contaminated by organic residues present in the FIB chamber. C is particularly strongly present in areas exposed to the electron beam during the EELS measurement^25,26^ (Areas 1 and 2, intense red square in Fig. 4) and its atomic rate reads 0.25 ± 0.05. Area 3 then exhibits the atomic rate of C of 0.08 ± 0.03 which is higher value than for EBL antenna. The atomic rate of O reads 0.05 ± 0.02 in Area 1 and 0.07 ± 0.02 in Areas 2 and 3 (i.e., in the antenna surroundings) which may indicate that Ti and Ga contaminants present in the surroundings are partially oxidized.

**Figure 5:**
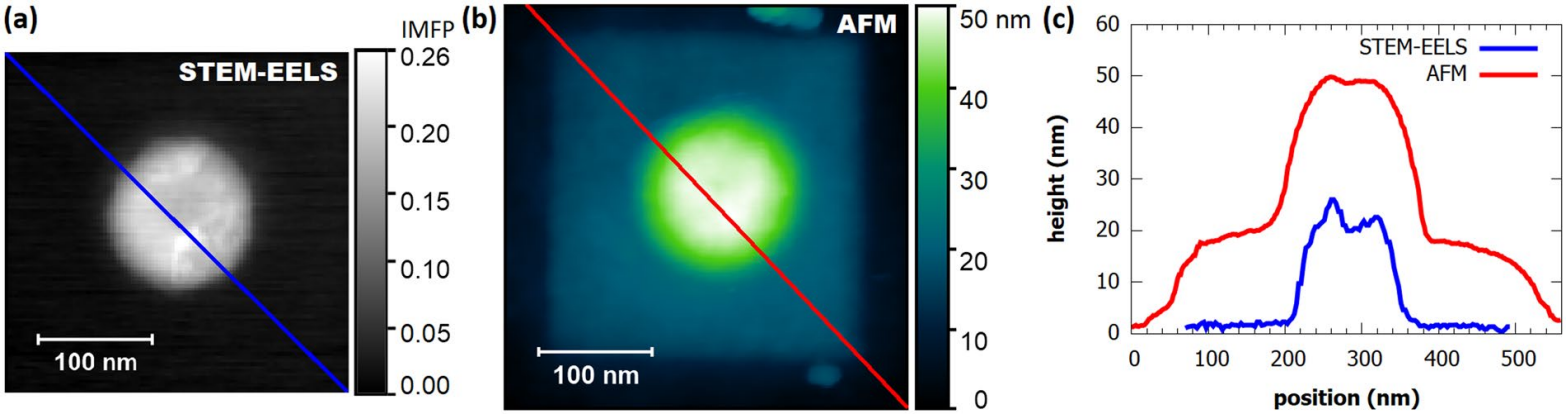
Thickness profile of a 140-nm FIB antenna and the hydrocarbon contamination on its surface. (**a**) Relative thickness (thickness in terms of IMFP) of the antenna retrieved by EELS. (**b**) Topography of the antenna including the hydrocarbon contamination measured by AFM. (**c**) Linear cross-sections of thickness profiles along the lines shown in (**a**) and (**b**). Relative thickness is recalculated to absolute thickness using the IMFP in gold of 113 nm. Blue line shows the thickness profile of the antenna determined by EELS, red line shows the thickness profile of the antenna and the contamination layer determined by AFM.

It is impossible to determine the thickness of hydrocarbon contamination layer on the FIB antennas using EELS because the contamination would further evolve during the measurement. Therefore, we have characterized surface topography of antennas by AFM measurement. Figure 5 compares the thickness of the 140-nm FIB antenna measured by EELS (assuming low hydrocarbon contamination) and surface topography measured by AFM after the EELS measurements (with hydrocarbon contamination being fully developed). The thickness of the hydrocarbon contamination layer is uniform over the whole area of EELS measurement (Areas 1 and 2) with the average thickness of (18 ± 5) nm determined as the height difference between Areas 2 and 3.

## Discussion

The choice of preferred fabrication technique should also consider the time and potential risks of the fabrication process. FIB preparation is simple and more straightforward as no chemistry is used and fast for individual antennas or small series of antennas. Moreover, FIB lithography exerts low mechanical strain and thus enables processing of pristine materials with low adhesion to the substrate such as chemically grown single-crystalline gold flakes. EBL preparation provides generally antennas with higher quality, but the lithographic process induces potential risks of damaging the sample as the wet chemistry is used. EBL is more time consuming than FIB if a small number of antennas is prepared, but much faster if a large array of antennas is fabricated. In such case, the volume of material to be removed by FIB is too large. Therefore, either of the techniques is suitable for a different class of targeted nanostructures. In general, when no specific issues apply, EBL shall be prioritized over FIB as it produces antennas with stronger plasmonic response and low contamination.

In conclusion, we have performed the comparative study of plasmonic antennas fabricated by EBL and FIB. We have demonstrated that the EBL antennas have better quality. First, they have rather homogeneous thickness profile with decent thickness fluctuation, and sharper edges. Second, they are cleaner, with a moderate contamination of organic origin evidenced by the presence of carbon and oxygen atoms with the total rate up to 20%. On the other hand, plasmonic antennas fabricated by FIB lithography have slightly dull edges and exhibit pronounced thickness fluctuation. They are also strongly contaminated not only by organic contaminants forming a continuous layer of the thickness of about 18 nm, but also by residues of FIB milling including implanted milling ions and atoms of the titanium adhesion layer, possibly oxidized. In consequence, the plasmonic response of the antennas characterized by EELS is considerably stronger and slightly better localized for the antennas fabricated by EBL than for the antennas fabricated by FIB. To conclude, while both techniques are suitable for the fabrication of plasmonic antennas, EBL shall be prioritized over FIB lithography due to better quality of the resulting antennas.

## Methods

### Membranes

We used standard 50-nm-thick silicon nitride membranes for TEM with the window size of 250 × 250 µm^2^ and frame thickness of 200 µm by Silson Ltd, UK.

### Electron beam lithography (EBL)

As the resist, we used a 70 nm thick layer of poly(methyl methacrylate) 679.02 all-resist dissolved in ethyl acetate. Exposition has been performed within Tescan MIRA3/RAITH SEM operated at 30 kV with the electron beam current of 100 pA and the dose of 320 µC/cm^2^. Exposed samples were developed in the developer AR 600-56 all-resist for 3 minutes, as the stopper we used isopropyl alcohol for 30 seconds. Afterwards, the sample was cleaned by demineralized water for 30 seconds. Lift-off was performed by 4-hour acetone bath applying mega sound for 1 hour finished by acetone stream wash. Finally, the sample was cleaned by isopropyl alcohol for 30 seconds, followed by cleaning using ethanol for 30 seconds and demineralized water for 30 seconds. To prevent the collective interaction of the antennas we fabricated individual isolated antennas with the distance between two nearby antennas of 3 μm.

### Metal deposition

We used electron beam evaporator BESTEC. Pressure during the deposition was in the order of 10^−5^ Pa and voltage was set to 8 kV. We deposited 5 nm Ti adhesion layer with the deposition speed of 0.05 nm/s and 25 nm Au with the deposition speed of 0.02 nm/s. Metal pellets were purchased from Kurt J. Lesker Company. Samples were rotated at 10 rounds per minute to ensure the homogeneity of the layers. Both samples were deposited during one session to have exactly the same metallic layer on both of them. During the deposition, the thickness of the layer was measured *in situ* by quartz crystal microbalance monitor.

### Focused ion beam (FIB) lithography

We used dual beam FIB/SEM microscope FEI Helios using gallium ions with the energy of 30 keV and ion beam current of 2.4 pA. We note that the energy (the highest available) and the current (the lowest available) are optimized for the best spatial resolution of the milling. The antennas were situated in the middle of a 2 × 2 μm^2^ metal-free square, which is perfectly sufficient to prevent their interaction with the surrounding metallic frame^36^.

### Electron energy loss spectroscopy (EELS)

EELS measurements were performed with TEM FEI Titan equipped with GIF Quantum spectrometer operated in monochromated scanning regime at 300 kV. Beam current was set to 0.8 nA and the FWHM of the ZLP was around 0.18 eV. We set convergence angle to 10 mrad, collection angle to 20.5 mrad, and dispersion of the spectrometer to 0.01 eV/pixel. We recorded EELS spectrum images with the size of 300 × 300 nm^2^ (100 × 100 pixels with the pixel size of 3 nm). Every pixel consists of 30 cross-correlated EEL spectra with the total pixel time of 20 ms. EEL spectra in Fig. 3 were integrated over the ring-shaped areas around the disc edge where the LSP resonance is significant (inner diameter about 50 nm, outer diameter about 200 nm) and divided by the integral intensity of the whole spectrum to transform measured counts to a quantity proportional to the loss probability. EEL maps were calculated by dividing the map of integrated intensity at the plasmon peak energy with the energy window of 0.1 eV by the map of the integral intensity of the zero-loss peak. Radial distributions of LSP resonances were calculated from the EEL maps by rotational averaging followed by the background subtraction (base level of the intensity in the EEL maps far away from the antennas).

### Energy dispersive X-ray spectroscopy (EDS)

EDS measurement was performed on TEM FEI Titan equipped with Super-X spectrometer operated in the scanning regime at 300 kV. Beam current was set to 2 nA. We recorded EDS spectrum images with the size of 600 × 600 nm^2^ (300 × 300 pixels with the pixel size of 2 nm). We integrated 100 images with the acquisition time of 10 µs per pixel. Spectrum images were post processed in Velox software. EDS maps in Fig. 4 show the net intensity (i.e. background subtracted and artifact corrected intensity). Table 1 shows the EDS quantification in atomic percents performed in Velox software using parabolic background model and Brown-Powell ionization cross-section model.

### Atomic force microscopy (AFM)

AFM measurement was performed using Scanning Probe Microscope Bruker Dimension Icon in the PeakForce Tapping mode, which enables larger control over applied force to prevent damaging the membrane^37^. The peak force setpoint was set to 5 nN, the scanning window was 400 × 400 nm^2^ (256 × 256 pixels with the pixel size of 1.6 nm).

### Simulations

Numerical simulations of EELS spectra were performed using the MNPBEM toolbox^22^ based on the boundary element method (BEM)^23,24^. The dielectric function of evaporated gold was taken from Olmon *et al*.^38^ and the dielectric function of the silicon nitride membrane was set to 4^39^. The titanium adhesion layer has been neglected in the simulations. For the calculations of spectra the electron beam was positioned 2 nm from the outer side of the antenna. Obtained loss probability density was recalculated to loss probability at 0.01 eV energy intervals (corresponding to the dispersion of the spectrometer in the experiment).

### Data availability

The datasets generated during and/or analyzed during the current study are available from the corresponding author on reasonable request.
